# Circadian Rhythms in the Telephone Calls of Older Adults: Observational Descriptive Study

**DOI:** 10.2196/12452

**Published:** 2020-02-25

**Authors:** Timothée Aubourg, Jacques Demongeot, Hervé Provost, Nicolas Vuillerme

**Affiliations:** 1 Orange Labs Chemin du Vieux Chêne Meylan France; 2 University Grenoble Alpes, AGEIS Grenoble France; 3 LabCom Telecom4Health University Grenoble Apes & Orange Labs Grenoble France; 4 Institut Universitaire de France Paris France

**Keywords:** outgoing telephone call, circadian rhythm, older adults, call-detail records, digital phenotyping, digital biomarkers, digital health, mhealth

## Abstract

**Background:**

Recent studies have thoughtfully and convincingly demonstrated the possibility of estimating the circadian rhythms of young adults’ social activity by analyzing their telephone call-detail records (CDRs). In the field of health monitoring, this development may offer new opportunities for supervising a patient’s health status by collecting objective, unobtrusive data about their daily social interactions. However, before considering this future perspective, whether and how similar results could be observed in other populations, including older ones, should be established.

**Objective:**

This study was designed specifically to address the circadian rhythms in the telephone calls of older adults.

**Methods:**

A longitudinal, 12-month dataset combining CDRs and questionnaire data from 26 volunteers aged 65 years or older was used to examine individual differences in the daily rhythms of telephone call activity. The study used outgoing CDRs only and worked with three specific telecommunication parameters: (1) call recipient (alter), (2) time of day, and (3) call duration. As did the studies involving young adults, we analyzed three issues: (1) the existence of circadian rhythms in the telephone call activity of older adults, (2) their persistence over time, and (3) the alter-specificity of calls by calculating relative entropy.

**Results:**

We discovered that older adults had their own specific circadian rhythms of outgoing telephone call activity whose salient features and preferences varied across individuals, from morning until night. We demonstrated that rhythms were consistent, as reflected by their persistence over time. Finally, results suggested that the circadian rhythms of outgoing telephone call activity were partly structured by how older adults allocated their communication time across their social network.

**Conclusions:**

Overall, these results are the first to have demonstrated the existence, persistence, and alter-specificity of the circadian rhythms of the outgoing telephone call activity of older adults. These findings suggest an opportunity to consider modern telephone technologies as potential sensors of daily activity. From a health care perspective, these sensors could be harnessed for unobtrusive monitoring purposes.

## Introduction

### Background

Circadian rhythms of activity—biological processes working on 24-hour cycles—can be used to represent relevant temporal markers in an individual’s life. They have two broad characteristics: (1) at the endogenous level, they are regulated by the brain’s suprachiasmatic nucleus (SCN), which can be considered, at least partly, as the individual’s biological master clock [1], and (2) at the exogenous level, regulation of the SCN is influenced by the *entraining power* of external *time givers*, known as zeitgebers [2]. Zeitgebers can be social, such as meal schedules [3] or work schedules [4], but they can also have a physical origin, as evidenced by the effect of light, exposure to which has a direct impact on an individual’s resting time [5].

In the field of health monitoring for older populations, the analysis of circadian rhythms of activity represents an increasingly important issue, one that may permit health care professionals to more adequately address their patients’ needs, treatments, and care [6]. Notably, by understanding their patients’ lifestyle habits, health care professionals may be better able to detect the possible occurrence of risky situations, such as a sedentary lifestyle [7], fall accidents [8], or sleep disturbances [9,10], that can have severe impacts on their health status. In practice, this monitoring is often challenging because the medical expert must be able to detect patients’ circadian rhythms of activity in a precise way. In the field of health care research, such promising approaches as actigraphy [11] have been proposed recently to tackle this issue. Interestingly, actigraphy has shown that it is possible to model older individuals’ gross motor activities by means of wearable sensors. Two recent reviews [8,12] on this subject notably described how this approach offers unprecedented opportunities to enhance traditional health care systems by using objective, inexpensive, and easy-to-use sensors [8]. These record individuals’ daily physical activities in a precise, real-time manner to help detect risky behaviors [7], signs of cognitive dysfunction [13], or severe events such as personal injury accidents [14], whose occurrence may increase with age.

At a clinical level, however, current opportunities for using actigraphy are limited. In older adults, although analysis of the circadian rhythms of their physical activities is well served by this approach, social activities have yet to be investigated. This issue is all the more important in aging populations because life—especially social life—is not purely physical, but is also subject to particular social events, such as a relative’s death [15] or the transition to retirement [16]; these events can be associated with the occurrence of severe health issues in older individuals, particularly illustrated by the phenomena of social isolation and depression [17]. It is also important to stress that social issues are not always reflected in an individual’s physical activity, but they remain significantly important to health, insofar as they can alter both the biological and social rhythms of life [18]. Thus, complementary methods are required to include the social aspects of older adults’ circadian rhythms of activity if we wish to monitor their health adequately.

### Prior Work

In today’s digital society, active and passive information collection methods, such as digital questionnaires or call-detail records (CDRs), generate vast amounts of data at high velocity from our telephones. This offers an unprecedented opportunity to capture and better understand the mechanisms of circadian rhythms in social activities [19]. Because the telephone has become such an integral part of our hyperconnected digital lives, as well as our children’s lives, telephone data may contain relevant information about the social interactions occurring throughout the day between individuals and their social networks.

In the field of health monitoring, exploiting these data could provide opportunities to enrich existing approaches that rely on monitoring physical activity by using objective, noninvasive data on social activity. This could also open up new research perspectives for innovation within traditional health care systems [20,21]. Interestingly, a recent literature review published in JMIR mHealth and uHealth [21] focused specifically on the use of CDRs provided by network communication operators. Notably, following their qualitative analysis of 46 full-text articles, the authors concluded on page 1 of the review that CDRs’ “potential to be used as a means of improving health care is increasingly promising” [21]. They further stressed several advantages of using CDRs in health research, including the ability to model individuals’ social behaviors by routinely and passively collecting data, thereby bringing robustness and effectiveness to surveys of individual telephone users. Moreover, because of the telephone’s ubiquity [19], the authors of the review stated on page 5 that using CDRs “does not preclude those from low socioeconomic groups” [21].

Other studies specifically examining the circadian rhythms of social activity have pointed to the possibility of estimating the expression of those rhythms by analyzing the CDRs of individuals’ telephone use [22-25]. A recent study from Aalto University in Finland provided a relevant methodology for estimating the circadian rhythms of social activity from the telephone communications of high school students [22]. Its authors analyzed an 18-month dataset for 24 adolescents, combining mobile telephone calls and questionnaire data to assess individual differences in the daily rhythms of mobile phone call activity. The results and their consistency demonstrated the existence of circadian rhythms in the outgoing telephone call activity in this young population, as did their alter-specificity (ie, in the way that callers, also named *egos*, place their calls with their recipients, also named *alters*, over the day). We should highlight that the studies mentioned above, which provided their participants’ ages, targeted groups consisting typically of students or young working adults [22-25]. Furthermore, as the authors themselves acknowledged [21] on page 5 of their review, one limitation of CDR analysis involves “the validity of study findings focusing on particular demographics, since the extent of group representation is not known” [21]. In other words, whether and how similar results to those reported for healthy young adults [22] could also be observed among other populations, including older adults, has yet to be established.

### Study Goals

This study was specifically designed to address the existence of circadian rhythms in the telephone calls of older adults at an individual level. In order to be able to compare this work with the existing literature [22], we focused on three specific issues in the following ways: (1) the existence of circadian rhythms, by computing the hourly ratio of outgoing telephone calls made by older individuals, (2) the consistency of such patterns, by evaluating their persistence over time, and (3) their alter-specificity, by using measures of relative entropy at a given time to analyze how callers allocated their communication time across their social network throughout the day (ie, the variety of alters communicating with their corresponding egos).

## Methods

### Data Collection and Volunteer Recruitment

Our dataset included 12 months of outgoing CDRs from 26 older volunteers in France: 20 women (77%) and 6 men (23%); median age 84 years (range 71-91). CDRs provided by the local network communication operator were collected from their personal telephones. Each telephone CDR contains the date; hour; source ID used (ego); destination user’s ID (alter); direction, which is established here as outgoing; and call duration in seconds. Individuals with several telephones registered with their network communication operator (ie, one or more landline telephones and/or one or more mobile phones) provided outgoing CDRs for all of them. Note that although CDRs contain both calls and text messages, this paper only selected telephone calls, so as to facilitate comparison with existing studies [22] in the literature. Also, once during the study, participants also completed a questionnaire about the contacts in their telephone social network. They classified each of their telephone contacts into one of five distinct social categories: family, friends, associations, health care professionals, and others.

This study and its corresponding experimental protocol were submitted to the French Data Protection Authority (Commission Nationale de l'Informatique et des Libertés [CNIL] registered data protection officer, France Telecom 2011 No. 44). All experimental methods were carried out in accordance with its regulations, written informed consent was obtained from all participants before data collection, and participant data were anonymized to ensure privacy.

### Data Preprocessing

As participants did not all enroll in the survey at the same time— thus, their dates of inclusion varied—the CDR dataset was filtered to select the time interval when the greatest number of older adults were actively participating. The CDR dataset was then preprocessed, following the method described by Saramäki et al [26], by selecting only the participants who used their telephones throughout the entire 12-month observation period. Therefore, the results cover a set of 21 individuals; see Table 1 for details.

**Table 1:** Structure of the call-detail records (CDRs) dataset before and after preprocessing.

Participant characteristics		Before preprocessing	After preprocessing
**Number of participants, n (%)**			
	Total	26 (100)	21 (100)
	Female	20 (77)	16 (76)
	Male	6 (23)	5 (24)
**Age in years**			
	Range	71-91	71-91
	Mean (SD)	84 (4)	83 (4)
Total number of outgoing calls		19,198	18,338
**Average number of calls per individual**			
	1st quartile	285	481
	Median	590	710
	3rd quartile	944	1096

### Data Analysis Procedures

#### Measuring the Circadian Rhythms of Outgoing Telephone Call Activity

We followed the descriptive approach designed by Aledavood et al [22], which consisted of calculating the circadian patterns of the outgoing telephone call activity of our older adult participants across the study’s entire 12-month dataset. This two-step process consisted of the following: (1) coarse-graining the time dimension into a unique day divided into 24 1-hour time slots and (2) calculating the average frequency of telephone calls for each time slot. We followed this approach at two distinct population levels: (1) the aggregate level, to obtain a concise overview of the dataset’s structure and trends and (2) the individual level, to obtain the circadian patterns of outgoing telephone call activity for each individual. Distinguishing the aggregate level from the individual level allowed us to avoid an ecological fallacy [27] when interpreting the results.

### Assessing the Consistency of Circadian Rhythms in Outgoing Telephone Call Activity

#### Overview

For assessing the consistency of circadian rhythms in outgoing telephone call activity, we followed an analytical approach based on the notion of persistence, which was first introduced for social signature analysis by Saramäki et al [26]. This notion was subsequently applied to studies estimating circadian rhythms [22-24]; the approach consists of comparing the stability of estimated patterns at distinct, successive time points by following three steps.

#### Step 1: Temporal Discretization

Data were coarse-grained as previously done by other authors [22]. Since the dataset contained 12 months of observations, they were split into three successive 4-month periods named T1, T2, and T3. Aggregating data into intervals of several months enhanced the robustness of persistence analysis by limiting the effects of short-term variations in call patterns [28]. This also reduced the probability of dealing with empty datasets.

#### Step 2: Circadian Rhythm Calculation

The circadian rhythm of each ego’s outgoing telephone call activity was calculated for periods T1, T2, and T3, as described in the previous section.

#### Step 3: Persistence Analysis

The persistence of each ego’s circadian rhythm was analyzed. This consisted of, first, calculating the stability of each ego’s call patterns between successive periods (T1, T2, and T3) and, second, comparing this stability with a reference scale measured by the square root of the Jensen-Shannon divergence (JSD) dissimilarity measure [27] (see Statistical Tools in the Methods section).

**Figure 1:**
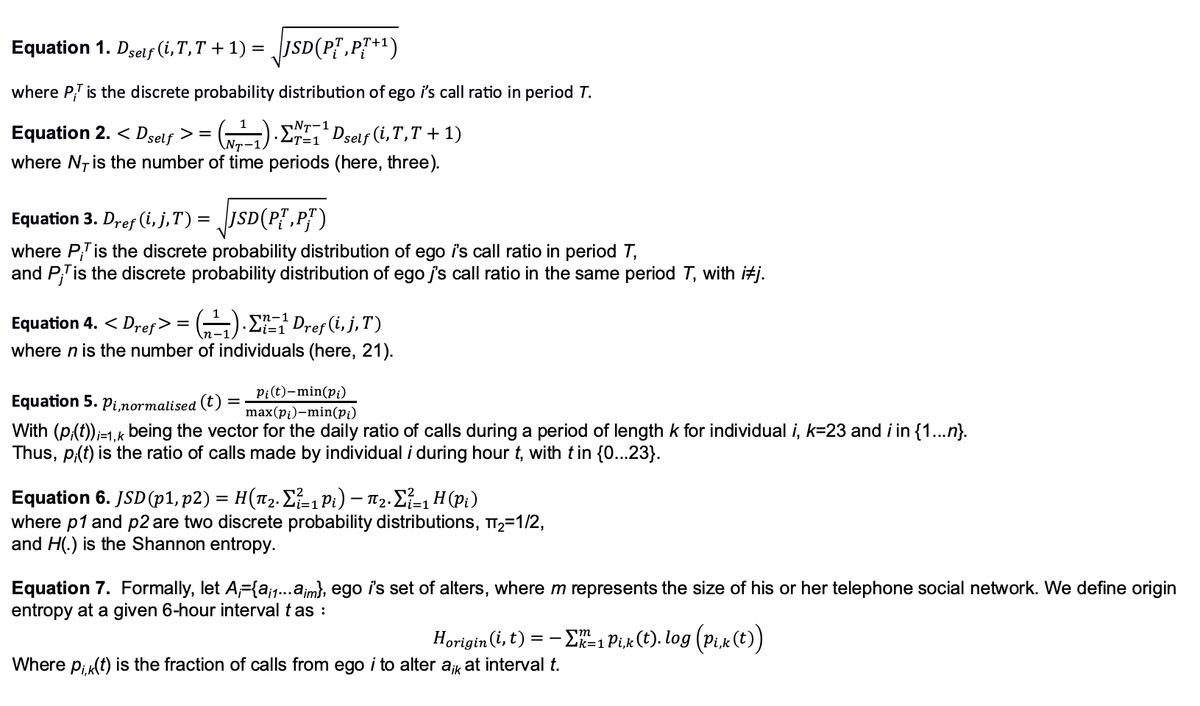
Equations used in the manuscript.

First, we note *D_self_* as a dissimilarity measure of the individual’s circadian rhythms between two successive periods (see Figure 1, equation 1). We note that <*D_self_*> is the average of *D_self,_* (see Figure 1, equation 2).

Second, a reference scale for further comparison was built for each ego, calculating the JSD between each ego and all the other egos in each period, T1, T2, and T3. We note *D_ref_* as a dissimilarity measure between two circadian rhythms of two distinct individuals in the same period (see Figure 1, equation 3). We note that <*D_ref_*> is the average of *D_ref_* (see Figure 1, equation 4).

Finally, the persistence of a given ego’s circadian rhythm was assessed by comparing his or her average call pattern over time with his or her average reference scale. Formally, an individual’s circadian rhythm can only be found to be persistent if, and only if, <*D_self_*>/<*D_ref_*><1.

### Measuring Alter-Specificity in Circadian Rhythms

The alter-specificity in the circadian rhythms of outgoing telephone call activity was investigated jointly using individuals’ CDRs and questionnaire data, as previously done by Aledavood et al [22]. Concretely, the three following points were analyzed according to the time of day: (1) the existence of specific hours used for communicating with a specific alter, (2) the ratio of outgoing telephone calls directed to each individual’s top 2 alters, and (3) the variation in telephone call durations between individuals and their social networks.

### Assessing the Existence of Specific Hours for Communicating With Specific Alters

For assessing the existence of alter-specificity in the circadian rhythms of outgoing telephone call activity, we made a two-step analysis of how each ego communicated with their alters throughout the day.

#### Step 1: Temporal Discretization

We began by coarse-graining the time dimension into four bins of equal duration—night (12 am-6 am), morning (6 am-12 pm), afternoon (12 pm-6 pm), and evening (6 pm-12 am)—for each ego and for periods T1, T2, and T3.

#### Step 2: Alter-Specificity Calculation

For each ego, we analyzed the alter-specificity of outgoing telephone call patterns over 24 hours during periods T1, T2, and T3, comparing the alter-structure of the estimated circadian rhythms with the alter-structure of a null model simulating total randomness in alter-specificity. To do this, we estimated the diversity of alters in each 6-hour bin for each ego and calculated the associated relative entropies (see Statistical Tools in the Methods section) during periods T1, T2, and T3. This calculation involved two steps: Step 2a and Step 2b.

#### Step 2a: Origin Entropy Calculation

First, we calculated each ego’s origin entropy *H_orig_* in each 6-hour bin for periods T1, T2, and T3.

#### Step 2b: Relative Entropy Calculation

Second, we obtained the relative entropy *H_rel_* by normalizing with *H_orig_* the average reference entropy <*H_ref_*>, which resulted from the null model. This null model works under the hypothesis that no specific times are associated with telephone calls to specific alters. Thus, a low relative entropy value, tending to 0, indicates that there is significant alter-specificity in the call pattern.

#### Measuring the Specificity of Outgoing Telephone Calls to the Top Two Alters

To investigate the rhythm of outgoing telephone calls from egos to their 2 favorite alters, we analyzed how the 2 favorite alters affected the circadian rhythm of the ego’s outgoing telephone call activity. To do this, we selected each ego’s 2 most frequently called alters for periods T1, T2, and T3 and for each of the four time bins. Next, we calculated the corresponding fraction of calls over each ego’s entire, 12-month, outgoing telephone call activity.

## Statistical Tools

### Normalization

To visualize the peaks and troughs of each individual’s telephone call activity, we normalized the daily ratio of their calls to be contained between 0 and 1, which enables us to avoid any excessive influence of extreme values on the graph’s colors. To this end, we used a unity-based normalization formula [29]. Formally, let *n* be the number of participants included in the study. Let (*p_i_*(*t*))*_i=_*_1,_*_k_* be the vector for the daily ratio of calls during a period of length *k* for individual *i*, with *k*=23 and the *i* range being {1...*n*}.

Thus, *p_i_*(*t*) is the ratio of calls made by individual *i* during hour *t*, with the *t* range being {0...23}.

Then, for each element *p_i_*(*t*) of *p_i_*, we note *p_normalised,i_*(*t*), its normalized value (see Figure 1, equation 5).

### Jensen-Shannon Divergence

The JSD is a measure of dissimilarity that compares two probability distributions; it is a symmetrical, finite-value version of the Kullback-Leibler divergence. Its square root can be used as a metric for measuring the distance between two probability distributions (see Figure 1, equation 6, for its definition in a discrete context).

### Entropy Measures

#### Overview

The entropy construct consists of three successive steps: (1) the origin entropy calculation measuring alter-specificity in the original dataset, (2) the reference entropy calculation measuring alter-specificity in a random dataset configuration [22], and (3) the relative entropy calculation standardizing the original entropy value in step 1 divided by the reference entropy value in step 2 [22]. The steps are discussed more precisely as follows:

1. The first step is the origin entropy calculation. This measures the diversity of the alters called by each ego in each of the four 6-hour intervals or bins during the day. The frequency of outgoing calls from each older adult to each of their alters is calculated separately for each 6-hour interval. Then, an entropy value—the origin entropy [22]—is calculated for each of these periods. This calculation enables us to quantify the proportion of distinct alters called by each older adult during corresponding periods.
2. The second step is the reference entropy calculation. This measures the diversity of alters called by each older adult during the day using a random configuration, named the null model, where there is no alter-specificity [22]. In the null model, the original call frequency patterns and the number of calls made to each alter are the same as in the original dataset, but associations between alters and the hour of calls are randomly shuffled so as to have no particular alter-specificity.
3. The third step is the relative entropy calculation. This compares the alter-specificity measurements obtained in step 1 with the alter-specificity measurements of their corresponding null models obtained in step 2.

In detail, this occurs for a given ego *i*, where *i* is between 1 and n, and n=21 is the number of participants.

#### Step 1: Origin Entropy

Formally, let *A_i_*={*a_i1_*...*a_im_*}, ego *i*’s set of alters, where *m* represents the size of his or her telephone social network. We define origin entropy at a given 6-hour interval *t*, as in equation 7 in Figure 1.

#### Step 2: Reference Entropy

We next define a null model, which simulates a system without a specific alter-structure. The number of calls from ego *i* to each alter is maintained, but the times of calls to their alters are shuffled in order to simulate randomness in each 2-week period, as done by Aledavood et al [22]. Next, for each 6-hour interval *t* of the shuffled dataset, we calculate the corresponding *H_origin_*(*i,t*) as defined in step 1. The reference entropy is obtained by iterating this process n=1000 times, as previously described, and calculating the corresponding average *H_origin_* for each time interval.

#### Step 3: Relative Entropy

To estimate the alter-specificity of the original system, we normalized the origin entropy of ego *i* at each time interval *t* by their previously calculated reference entropy:

H_relative_(i,t)=H_origin_(i,t)/H_reference_(i,t)

## Results

### There Are Circadian Rhythms in the Outgoing Telephone Call Activity of Older Adults

**Figure 2:**
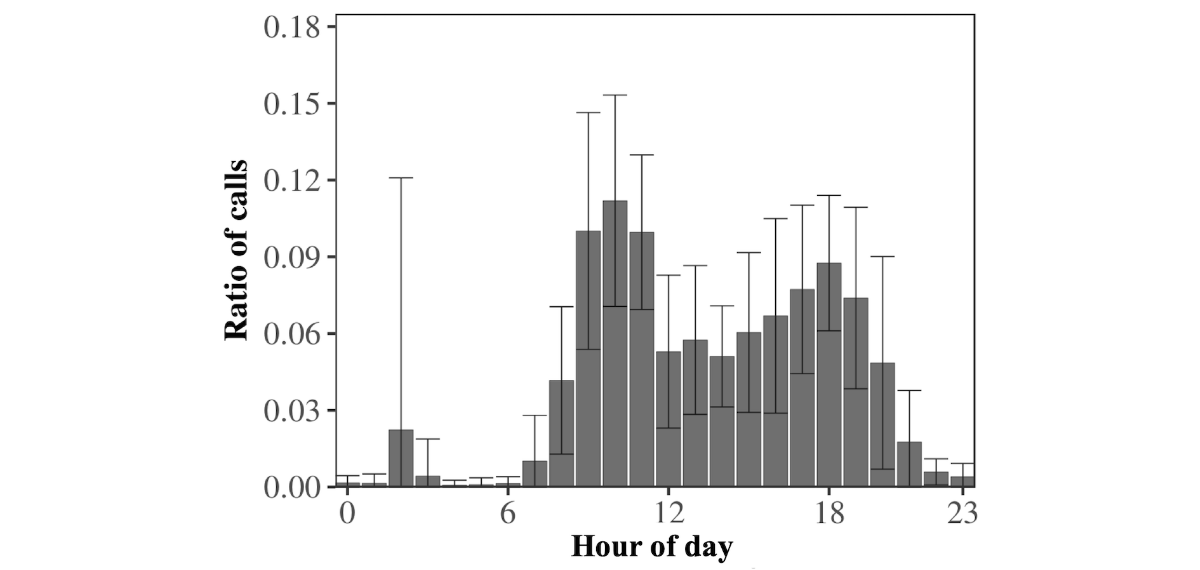
Aggregate-level circadian pattern of the outgoing telephone call activity of older adults. Bars represent the average hourly frequency of outgoing telephone calls. Error bars represent the hourly standard deviations. The wide range of the 5% error bars indicates that average outgoing telephone call activity differed significantly between individuals, especially at 2 am.

This study’s results suggest the existence of circadian rhythms in the outgoing telephone call activity of older adults at both the aggregate and individual levels. At the aggregate level, Figure 2 shows that the population’s average circadian pattern of outgoing telephone call activity was marked by two distinct peaks: one in midmorning, around 10 am, and one toward the end of the day, around 6 pm. These twin peaks were separated by a period of low activity in the afternoon, beginning at around 2 pm. The first activity peak, in midmorning, was higher than a second peak that occurred at the end of the afternoon. Interestingly, there was a small nocturnal activity peak at around 2 am, which was unusual with regard to the dataset’s marked diurnal pattern.

**Figure 3:**
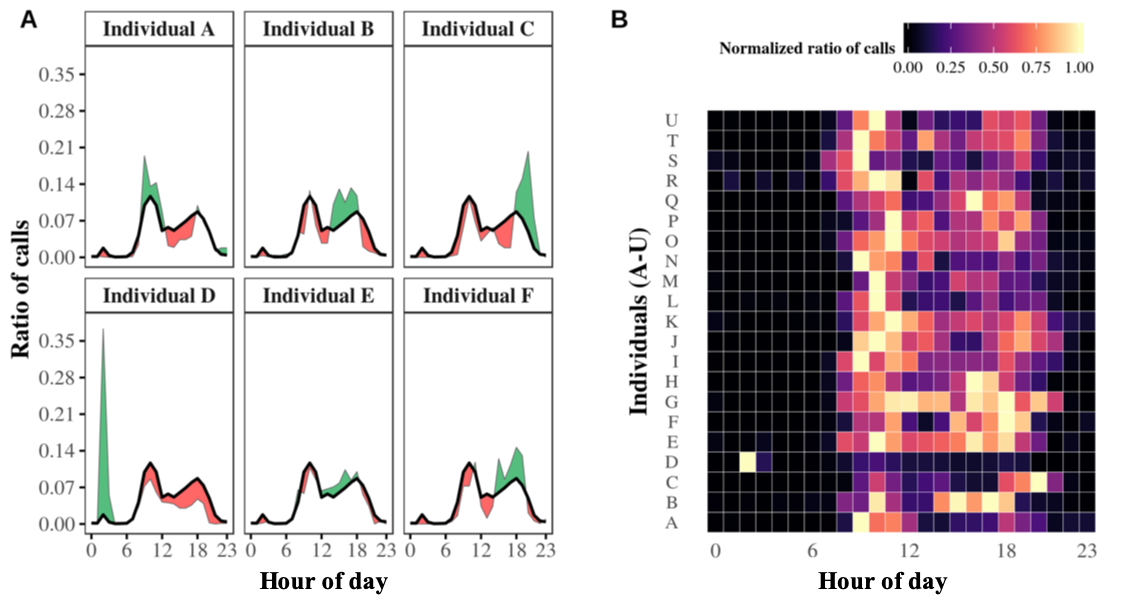
Circadian patterns of outgoing telephone call activities at the individual level. Panel A. Individual circadian patterns of the outgoing telephone call activity profiles of 6 distinct egos. Each small graph shows a specific ego. The black line represents the average circadian pattern of outgoing telephone calls for the population, whereas the individual’s circadian pattern of calling is represented by a green or red area indicating higher or lower outgoing telephone calls than the average. Panel B. Heat map showing 21 individual outgoing telephone call patterns. The entire population’s normalized individual circadian patterns are shown aggregated onto one heat map. Each horizontal strip summarizes the circadian pattern of one ego, associating each hour of the day with the corresponding ratio of outgoing telephone calls in appropriately colored boxes. The greater the ratio of outgoing telephone calls, the brighter the corresponding box.

First observations were subsequently refined by zooming into the individual level. The results also highlighted the circadian rhythms of outgoing telephone call activity for each participant separately. Figure 3, A illustrates the different outgoing communication patterns of a sample of 6 distinct egos from night to evening. Individuals A and E showed a preference for calling in the morning, whereas individuals B, C, and F preferred the afternoons or evenings. In particular, we noted a potential atypical pattern illustrated in Figure 3, A. Individual D displayed a prominent and atypical nocturnal calling activity at around 2 am and, interestingly, it was directed to one specific alter.

To put these examples back into their context, we normalized each ego’s hourly call frequencies between 0 and 1, where 1 was assigned to the maximums reached and 0 to the minimums reached (see Statistical Tools in the Methods section). This standardization enables us to avoid the visual distraction and influence of extreme values, such as any particularly high peaks in telephone call activity, when mapping frequencies on a graph. Thus, the results are displayed on the comparative heat map of Figure 3, B, synthetically illustrating the existence of the older adults’ various circadian rhythms, from night to evening. It seems that individual D was the only one leading a nocturnal lifestyle, but this observation explains the small, atypical nocturnal activity peak occurring at around 2 am at the aggregate level (see Figure 2).

### Circadian Rhythms of Outgoing Telephone Call Activity May Be Consistent Among Older Adults

**Figure 4:**
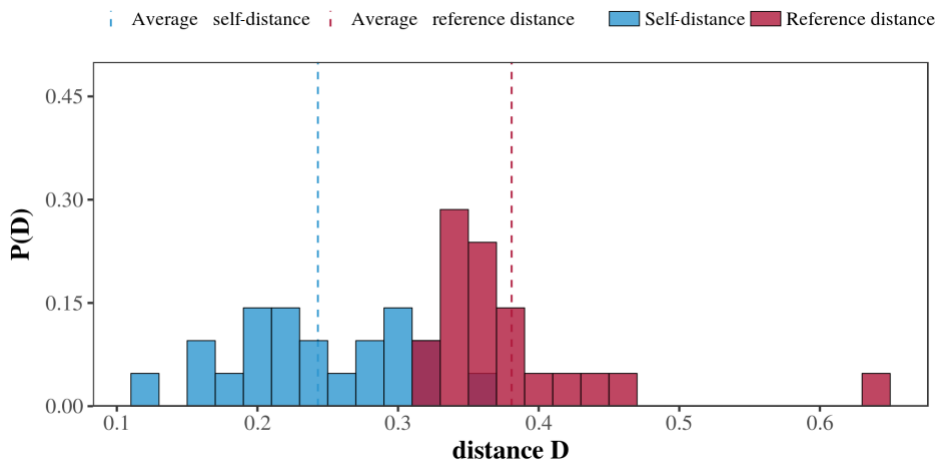
Averaged persistence histogram. Red bars represent average reference distances and blue bars represent average self-distances for all the egos. Blue and red dashed lines represent the average self-distance and reference distance for the entire population, respectively.

The consistency of the circadian rhythms calculated above was confirmed by our persistence analysis (see Methods section for details). Figure 4 displays the results of this analysis for all the egos. The average self-distance *D_self_* of all the egos is clearly lower than their average reference distance *D_ref_*. More precisely, on average, <*D_self_>*=0.24 (SD 0.06), whereas <*D_ref_>*=0.38 (SD 0.07). Indeed, all the egos’ self-distances were lower than their reference distances, which implies that their circadian patterns tended to retain their shape over time. These results were also confirmed using Aledavood et al’s more statistical approach [22]. We compared each ego’s successive circadian rhythms of outgoing telephone call activity by using a Kolmogorov-Smirnov comparison test. We obtained *P* values greater than .05 in all 42 cases (21 participants; *D_self_* and *D_ref_*), implying that the case for suggesting similarity between successive egos’ patterns cannot be rejected. We also repeated the analysis by changing the JSD measure for a classic Euclidean distance (L2), as proposed by Aledavood et al [22], and drew a similar conclusion. See Table 2 for details.

**Table 2:** Summary of the averaged √JSD^a^ and Euclidian distance (L2) metrics for the entire population’s references and self-distances.

Measures	√JSD distance	L2 distance
Self-distance, mean (SD)	0.24 (0.06)	0.14 (0.04)
Reference distance, mean (SD)	0.38 (0.07)	0.23 (0.07)

^a^JSD: Jensen-Shannon divergence.

### Alter-Specificity Is Indeed Evidenced in the Circadian Rhythms of Older Adults’ Outgoing Telephone Call Activity

**Figure 5:**
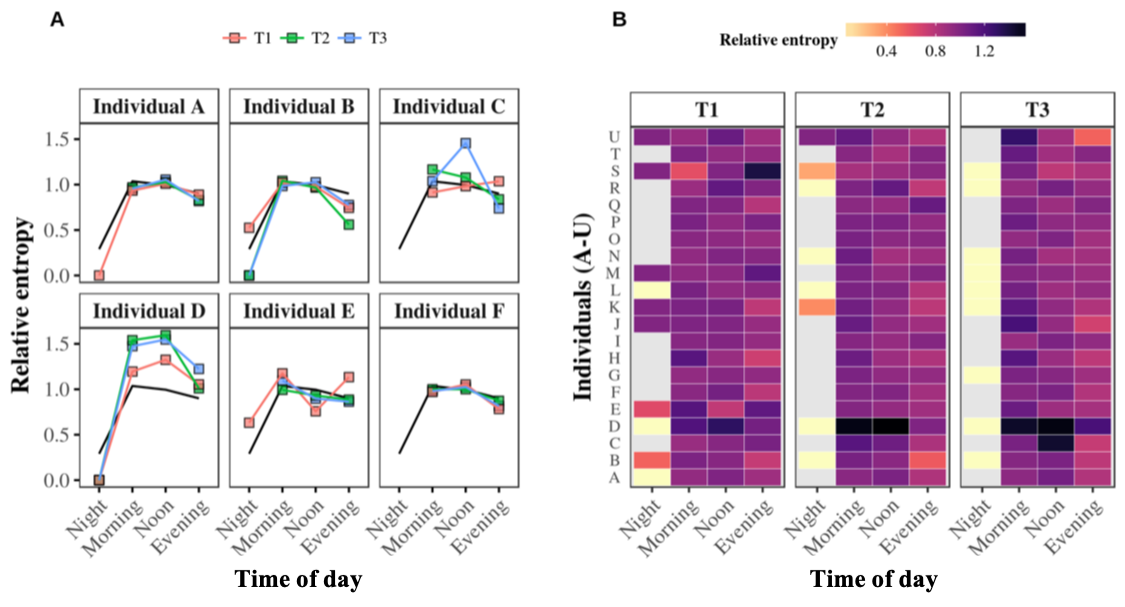
Relative entropy values at the individual level. Panel A. The relative entropy values of the same 6 individuals described in <xref rid="figure2" ref-type="fig">Figure 2</xref> for the night, morning, afternoon, and evening are calculated for the first, second, and third 4-month periods: T1 (red curve), T2 (green curve), and T3 (blue curve), respectively. The black curve represents the whole population’s average relative entropy value over the three periods. Panel B. The heat map summarizes all the relative entropy values of all 21 egos. The lower or higher the relative entropy, the brighter or darker the color assigned to it, respectively. Missing values are assigned a grey color.

Overall, these results suggest the existence of alter-specificity in the circadian rhythms of older adults’ outgoing telephone call activity. This alter-specificity is reflected by the relative entropy calculation, which indicates whether communication with specific alters takes place at specific times of day. Figure 5, A illustrates the relative entropy results calculated for the 6 egos in Figure 3. In general, in this figure, the average entropy of the population (black line) reveals that egos tend to have low entropy values in the evening and at night but higher entropies in the morning and afternoon. The results also imply that older adults’ communications tend to be more focused on specific alters in the evening. The figure nevertheless shows that this trend does not hold for every participant, for instance, ego E.

These examples are put into context in Figure 5, B, which puts all the individuals’ entropy values for periods T1, T2, and T3 onto one heat map. Although some egos clearly only made outgoing telephone calls to a narrow range of alters, this observation cannot be generalized over our entire population of older adults. Moreover, although a relative entropy value tending to 1 indicates a broad diversity of alters, as per Aledavood et al [22], there are specific contexts in which a high relative entropy value does not necessarily reflect the diversity of alters in the egocentric networks of our older individuals. The appearance of such a context depends on the null model’s ability for generating significant randomness (see Statistical Tools in Methods section for details). If we consider an older individual whose entire nocturnal outgoing telephone call activity consists of a single call, this context should be evidenced by a low origin entropy value tending to 0. Consequently, a null model with a random structure should also logically provide a low reference entropy value tending to 0. There is no way to break down such detail because, by definition, the null model affects individuals’ time associations but preserves the number and time frequencies of calls. Thus, normalizing such a small origin entropy value by such a small reference entropy value, even when averaged, automatically makes the resulting relative entropy value skyrocket toward 1. A similar mathematical risk is attenuated by just a few more calls, but it will still exist if the ego’s overall outgoing telephone call activity is predominantly aimed at a few distinct alters. Thus, this paper’s notion of relative entropy must be considered accurate for making decisions about alter-specificity in a specific time interval. This means a high relative entropy tending to 1 does not necessarily imply the existence of a high diversity of alters in the ego’s network. Instead, it does not let us conclude that there is a high degree of alter-specificity. Additionally, Aledavood et al’s description of relative entropy results [22] also points out other originalities concerning this notion, such as its ability to exceed 1 in particular contexts.

**Figure 6:**
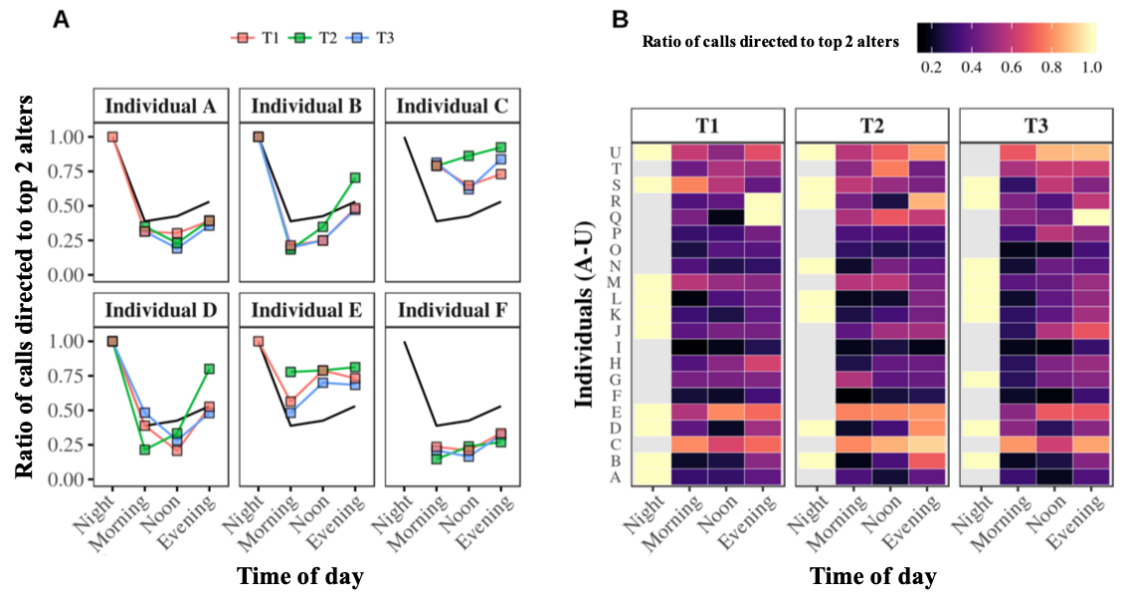
Fraction of outgoing telephone calls to top 2 alters at the individual level. Panel A. Each graph displays one participant’s fraction of calls to their top 2 alters during the night, morning, afternoon, and evening, calculated for periods T1 (red curve), T2 (green curve), and T3 (blue curve). The black curve represents the average fraction of calls to the top 2 alters of the entire population over the three periods. Panel B. These heat maps summarize the fractions of outgoing telephone calls going to the top 2 alters of each of the 21 egos. High fractions are assigned brighter colors, whereas low fractions of calls are assigned darker colora. Missing values are assigned the color grey.

The results also suggest that the alter-specificity evidenced above by the relative entropy may be associated with the older adult’s 2 most frequently called alters. For example, Figure 6, A shows the fraction of calls to the top 2 alters averaged at the population level for the whole year (black line) against those of the same 6 egos in Figures 2 and 4, averaged for periods T1, T2, and T3. The population pattern in this figure reveals that the fraction of calls to the top 2 alters reaches a maximum in the evening and at night, and it decreases in the morning and afternoon. It also seems proportionally inversed with the relative entropy variation (see Figure 5, A). Interestingly, the aggregation of the results of each individual’s top 2 alters in Figure 6, B’s heat map seems to confirm this trend. We further evaluated this trend by calculating the correlation between relative entropy and the fraction of calls to each ego’s top 2 alters during each 6-hour bin. Of 21 egos, 11 (52%) showed a significant negative correlation between entropy and the fraction of calls to their top 2 alters (*P*<.05); the 21-ego population average was close to -0.87. These results confirmed that for 11 of the 21 egos, a low entropy value correlated with a high fraction of their calls to their top 2 alters. No conclusions could be drawn for the other 10 egos.

**Figure 7:**
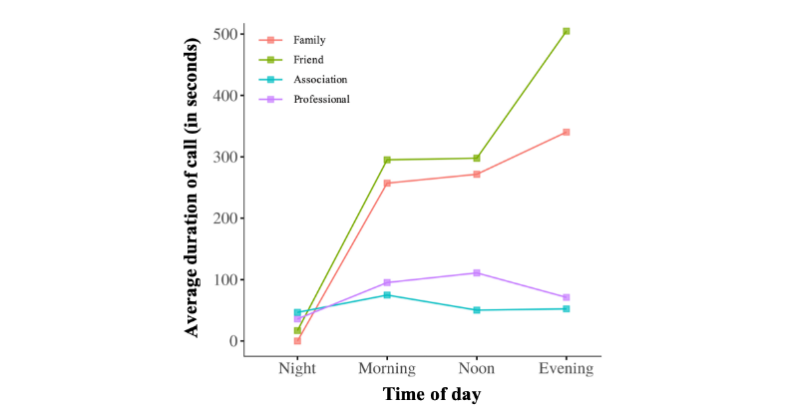
Aggregate-level average call duration throughout the day. Curves represent average call durations from egos to their families (red curve), friends (green curve), health care professionals (purple curve), and clubs or associations (blue curve).

Interestingly, by using questionnaire data, as did Aledavood et al [22], we recorded additional information about the kinds of telephone communications occurring between egos and alters throughout the day, according to their social relationship and the telephone call duration parameter. Two insights from these results clearly stand out in Figure 7: (1) the duration of outgoing telephone calls tended to be at a minimum at night and then increased throughout the day to the evening and (2) on average, egos communicated for longer with their restricted social network (ie, family and friends) than with their wider social network (ie, associations and health professionals). The longest telephone calls between older individuals and their friends and family occurred in the evenings. On the contrary, the duration of telephone calls to health care professionals and associations decreased throughout the day. This difference was not surprising. Indeed, in France, associations and health care professionals are not typically available by telephone after 5 pm.

## Discussion

### Principal Findings

Because studies to date on the circadian rhythms of telephone telecommunications at the individual level have focused especially on young individuals [22-25], we investigated whether similar results could be observed among older adults. We focused on a combination of CDRs and questionnaire data to examine a small sample of participants aged 65 years or older (n=21). Our analysis was based on CDRs of their outgoing calls over 12 consecutive months and was restricted to three specific telecommunication parameters: (1) the telephone call recipient (alter), (2) the moment in time, and (3) telephone call duration. Additionally, we evaluated questionnaire data collected from participants on their level of closeness with their alters, classified into the four broad categories of (1) friend, (2) family, (3) association, and (4) health care professional. To be able to compare our results with those in the existing literature, we used the methodology proposed by Aalto University [22] and focused on three specific issues: (1) the existence, (2) the consistency, and (3) the alter-specificity of the circadian rhythms of outgoing telephone call activity among older adults.

Overall, results showed that older adults had their own specific circadian rhythms of outgoing telephone call activity; salient features varied between individuals, with different preferences involving calling from morning to night and throughout the day (see Figure 2). It was subsequently demonstrated that these circadian rhythms of outgoing telephone call activity were also consistent, as reflected by their persistence over time (see Figure 3). Our investigation of the alter-specificity of these rhythms showed that they seemed partly structured by how older adults allocated their communication time among their social network. Indeed, on average, the relative entropy calculations and the older adults’ ratios of calls to their top 2 alters (see Figures 4 and 5) suggested that, although the evening and night were mostly reserved for a few specific alters, the morning and afternoon were more likely to be used for calling their wider social network. In addition to this temporal factor, the nature of telephone communications also seemed to be influenced by the social relationships between egos and their alters. Indeed, the average call duration in this older adult population was much higher for conversations with friends or family than with contacts in associations or health care. Call duration also varied throughout the day, with shorter calls in the morning and afternoon and longer telephone conversations occurring in the evening and at night.

Taken together, these findings provide the first support for the hypothesis that telephone CDRs can provide fine-grained data whose analysis can help to estimate older individual’s circadian rhythms of telephone call activity. These circadian rhythms notably influenced the way individuals interacted with their social network over 24 hours, which may have been a demonstration of appropriate social sensibility [30]. Sensibility is linked to the concept of intentionality [30] and may rely, in this paper, on the older adult’s ability to efficiently organize and prioritize their communication time throughout the day. This efficient organization may be reflected by at least two evident observations: (1) telephone calls to medical professionals generally took place early in the day, when they were most readily available, instead of in the evening, an unfavorable time for business interactions, and (2) calls to friends and family generally occurred in the evening, when they were more likely to be available and responsive. For retired people, this second point could reflect the fact that family and friends have social constraints of their own, such as work schedules, household tasks, or childcare duties, making them unwilling to accept being disturbed as their days begin. Thus, based on social sensibility, particular changes in the circadian rhythms of telephone call activity, such as persistent attempts to call friends and family in the morning and health care professionals in the evening, may be representative of a behavioral drift. This drift may be a valuable indication of a disruption to their ability to organize or prioritize social calls throughout the day. Three points thus stand out:

1. At the social level, these findings suggest that the results of recent studies on young populations could be extended to older ones [22]. A relevant perspective for future studies surely lies in increasing the sample sizes of the populations studied to compare these results against findings with greater statistical power.
2. At the health monitoring level, telephones have the potential to become sensors of older adults’ daily social activities. The valuable data they generate about older adults’ social lives, such as how they communicate with relatives and health care professionals over time, promises future innovative monitoring methods. Information collected objectively and noninvasively could provide health care professionals with behavioral insights about their patients in the form of alerts. Direct reflections of an individual’s telephone call activity [31] may help those professionals to better prevent the occurrence or worsening of certain severe health issues, such as social isolation or depression [17]. Clinicians might also use older individuals’ circadian rhythms of social activity or social sensibility timing to determine the best time of day to send health care prompts and information or set up individual telephone consultations by targeting hours when patients are more inclined to respond [32]. Enhancing the synergies between health care professionals, patients, and new monitoring technologies [33] could help to reinforce preventive, participative, pluri-expert, predictive, and personalized (5P) medicine [34].
3. At the clinical level, the information on social activity provided by CDRs could be harnessed to complement existing innovative approaches to monitoring physical activity, such as actigraphy [11,35], to provide a complete picture of older adults’ daily activities. They could also enrich traditional methods of clinical practice, such as health questionnaires requiring the patient’s active participation at a given time, by means of passive daily data collection that requires no supplementary effort from the patient.

It is also important to mention that in the field of health research, the methods designed by Aledavood et al [22] and used in this paper could provide a methodology for measuring the quality of CDRs. This is a challenging point given that the numerous studies investigating correlations between specific health issues, such as depressive symptoms [30], and daily behaviors measured using telephone data have (1) shown significant but contradictory results [36] and (2) not yet had their discrepancies explained. Hence, by assessing the consistency of the observed phenomena in such studies, persistence analysis could be an interesting means of addressing these two points together.

Telephones and the methodology used in this paper may provide an innovative, relevant future direction for the field of health monitoring of older adults. However, before classifying the telephone as a valid clinical sensor of social activity in older populations, a number of caveats and limitations should be considered.

### Limitations

First, any rapid, straightforward generalization of our results should be avoided because of the small sample size of 21 older adults remaining after the data preprocessing step. This small sample means that our results could suffer from an as yet unquantified selection bias. Our interest in working with a small sample of individuals lies in the opportunity to collect, monitor, and analyze individual datasets of significant richness, mixing behavioral observations with social information over a long period of time. The added value of a study with such a dataset configuration is not the generalization of results but rather the acquisition of significant insights for future studies of big data [22].

Because we followed our preprocessing step precisely [26]—also used for circadian rhythm estimation methodology in telephone calls [22]—our data may have been open to another selection bias. Of our 26 initial participants, only those who had been active throughout the observation period were selected, as in previous studies [26]. Individuals whose telephone call activity ceased for any given reason were excluded from this study. On the one hand, filtering was justified because it separated viable participants from those who had encountered specific problems, such as changing telephones, changing telecommunications operators, or a desire to withdraw from the study for personal reasons. On the other hand, the preprocessing step could prove problematic in some cases because a lack of information could be a source of information in itself [37]. This is the case when missing values result from a change in individual behavior caused by a given event or a set of events, such as an accident, a disease, anxiety, depression, or social isolation. Consequently, excluding individuals from the study because of an absence of telephone call activity automatically introduced a clear selection bias into our analysis. Thus, we have to mention that our results only stand for older individuals who showed telephone call activity throughout the entire duration of the study.

Finally, our results did not indicate that the circadian pattern of outgoing telephone call activity of any individual was a manifestation of their complete inherent circadian rhythm. Telephone call patterns should only be interpreted as an insight into an individual’s overall behavior, one that allows us to draw a first draft of their true inherent circadian rhythm. In today’s hyperconnected society, individuals can have social interactions through multiple communication media: telephone calls, text messages, or virtual social networks. A relevant way to enhance the robustness of circadian estimation could be to combine outgoing telephone call patterns with other sources of data (eg, incoming calls, text messages, and location data) and, more generally, to attempt a multidimensional analysis based on active and passive temporal data from telephones [38]. In the last few years, combining such data at the individual level has led to the birth of totally new domains of research [39]. For instance, in health, the digital phenotyping concept [40] attempts to surpass the expectations of classic individual actigraphy by mixing active and passive data generated by everyday tools like mobile phones [41]. Actigraphy typically presents such obvious disadvantages as the perceptions that it is both too invasive for patients with regard to medical protocols and too vague with regard to their social situation. Using a mobile phone as a multi-sensor can eliminate the costs of multiple external actigraphy devices, and this everyday object eases patients’ concerns about health care monitoring. A recent study [42] even reported that patients were more likely to share their suicidal thoughts with a mobile phone app than with their own psychiatrist.

### Perspectives for the Future

As other authors have mentioned [22], it would be interesting for future studies to compare individuals’ incoming and outgoing communications with their social network. This could be all the more important in health care contexts given that disruptions to an individual’s social interactions may be the sign of such severe health issues as depression [17]. With this in mind, we have immediate plans for a follow-up study comparing incoming and outgoing telephone calls in the hope of revealing any synchronous or asynchronous phenomena at the individual level.

## Abbreviations

5P: preventive, participative, pluri-expert, predictive, and personalized
CDR: call-detail record
CNIL: Commission Nationale de l'Informatique et des Libertés
JSD: Jensen-Shannon divergence
L2: Euclidean distance
SCN: suprachiasmatic nucleus
T1: first 4-month period
T2: second 4-month period
T3: third 4-month period
